# Determination of the self-attenuation based on the sample composition in gamma-ray spectrometry of ^210^Pb: requirements for the scope of chemical analyses

**DOI:** 10.1007/s10967-016-5054-4

**Published:** 2016-10-03

**Authors:** Paweł Jodłowski, Przemysław Wachniew, Jakub Nowak

**Affiliations:** Faculty of Physics and Applied Computer Science, AGH University of Science and Technology, Al. Mickiewicza 30, 30-059 Kraków, Poland

**Keywords:** Gamma-ray spectrometry, Self-attenuation, ^210^Pb, Chemical composition, Monte Carlo simulation

## Abstract

In the presented paper analysis of sensitivity of self-attenuation correction *C*_*s*_ to the accuracy of chemical composition analysis is presented. The analyses were done by means of Monte Carlo simulation for cylindrical samples and for four sample materials: peat, water, ash and soil. For each of these materials the major elements were selected whose determination in the analysed material is necessary. For the remaining elements threshold levels of their concentration were determined—if expected element concentration in a sample exceeds this value, its determination is indispensable, assuming the accuracy of *C*_*s*_ determination at 3 %.

## Introduction

Naturally occurring radionuclide ^210^Pb is commonly used in studies of processes associated with sediment accumulation in surface water bodies, river valleys and hill slopes. The most common application of ^210^Pb is connected with determination of sediment age and tracing of eroded soils and sediments. Gamma-ray spectrometry method enables accurate ^210^Pb determination but the key issue for measurement accuracy, especially for low-energy radiation emitted by ^210^Pb (46.5 keV), is determination of self-attenuation correction accounting for photon attenuation in volume sample. If the sample and the standard are handled in the same geometric setup this correction with respect to the standard (calibration source) is evaluated as a ratio of the detector efficiency for the standard *ε*_*c*_ to the detector efficiency for the sample *ε*_*s*_:

1 $$C_{s} (E) = \frac{{\varepsilon_{c} }}{{\varepsilon_{s} }}.$$

The *C*_*s*_ correction is significant for measurements of low energy gamma radiation. In such cases its determination is particularly troublesome (cf. [1]). This difficulty stems from the fact that the mass attenuation coefficient for low energies differs considerably between elements, therefore the impact of the chemical composition on attenuation properties of the material studied is decisive.

Two practical approaches have been developed to cope with that problem. The *C*_*s*_ correction is either determined experimentally or calculated on the basis of the known chemical composition and density of the material.

Usually the transmission method proposed by Cutshall [2] is used for the experimental evaluation of *C*_*s*_. This method combines the values of the linear attenuation coefficient obtained by the transmission measurement with the so-called self-attenuation equation for cylindrical samples. However, the simplified formula used in this method is questioned by many authors who observed that its application leads to systematic errors and proposed modifications [3–5].

As advanced methods of chemical analyses (e.g. fluorescence analysis, mass spectrometry) have become more widely available, some papers confirm the viability of evaluating the *C*_*s*_ correction based on sample chemical composition [6–8]. Methods used in computing the *C*_*s*_ correction on the basis of the chemical composition include: application of self-attenuation equation, the Debertin method [9] and Monte Carlo simulations.

In this paper the analysis of sensitivity of *C*_*s*_ estimation to the accuracy of chemical composition analysis is presented. In order to determine *C*_*s*_ correction it is necessary to know the chemical composition of the analysed sample, however, there is no need to determine all elements in a sample. The aim of this work is to identify the minimal scope of chemical analyses and the particular elements whose determination is indispensable.

## Experimental

In order to evaluate sensitivity of self-attenuation correction *C*_*s*_ to the accuracy of chemical composition analysis the ratio *C*_*s*_ */C*_*s*0_ was determined for different elements, where *C*_*s*0_ is the self-attenuation correction for a given material (e.g. peat—Fe concentration 5 %, cf. Table 1) and *C*_*s*_ is correction for the same material with a different concentration of this element (e.g. 6 % Fe). The *C*_*s*_ */C*_*s*0_ ratio equals

2 $$\frac{{C_{s} (E)}}{{C_{s0} (E)}} = \frac{{\varepsilon_{c} /\varepsilon_{s} }}{{\varepsilon_{c} /\varepsilon_{s0} }} = \frac{{\varepsilon_{s0} }}{{\varepsilon_{s} }},$$

where *ε*_*s*0_ is detector efficiency for the given material and *ε*_*s*_ is efficiency for the same material with different concentration of the element. As can be seen from (2), value of *C*_*s*_ */C*_*s0*_ does not depend on the detector efficiency for the material of the standard (cf. Eq. 1).

**Table 1:** Threshold concentrations for the studied materials and elements

		Peat^a^	Water^b^	Ash^c^	Soil^d^
		*μ*_*l*_ = 0.1300^e^	*μ*_*l*_ = 0.2378	*μ*_*l*_ = 0.5129	*μ*_*l*_ = 0.6242
		*C*_*s*_ = 0.880^f^	*C*_*s*_ = 1.000	*C*_*s*_ = 1.344	*C*_*s*_ = 1.486
C	*μ*_*m*_ = 0.1927^e^	MajEl (60 %)	60 % (0 %)	25 % (0 %)	20 % (1 %)
O	*μ*_*m*_ = 0.2251	MajEl (18 %)	MajEl (89 %)	MajEl (48 %)	MajEl (50 %)
Al	*μ*_*m*_ = 0.4188	45 % (3 %)	15 % (0 %)	40 % (13 %)	35 % (7 %)
Si	*μ*_*m*_ = 0.5049	MajEl (12 %)	10 % (0 %)	MajEl (21 %)	MajEl (34 %)
Ca, K	*μ*_*m*_ = 1.224^g^	9 % (2 %)	*2.5* *%* (0 %)	*11* *%* (7 %)	*4* *%* (1 %)
Fe	*μ*_*m*_ = 2.382	*7* *%* (5 %)	*1.1* *%* (0 %)	*7* *%* (5 %)	*5* *%* (4 %)
Pb	*μ*_*m*_ = 9.682	0.7 % (0 %)	0.25 % (0 %)	0.3 % (0 %)	0.25 % (0 %)

Threshold values in italics concern elements whose determination in the analyzed material is necessary if the expected element concentration in a sample exceeds threshold value and the underlined threshold values concern elements whose determination is not necessary. Numbers in brackets represent the baseline concentrations of elements (details cf. section “Experimental”)

*MajEl* major elements

^a^Exemplary peat; chemical composition: 60 % C, 18.5 % O, 11.5 % Si, 5 % Fe, 3 % Al, 2 % Ca; density 0.35 g/cm^3^

^b^Water; density 1.00 g/cm^3^

^c^Typical ash; chemical composition: 47.9 % O, 20.7 % Si, 12.9 % Al, 7.4 % Ca, 5.3 % Fe, 3.2 % Mg, 2.6 % S; density 1.0 g/cm^3^

^d^“Average” soil; chemical composition: 50 % O, 34 % Si, 7 % Al, 4 % Fe, 1 % C, 1 % Ca, 1 % K, 0.7 % Na, 0.6 % Mg, 0.5 % Ti, 0.1 % N, 0.1 % P; density 1.4 g/cm^3^ [16]

^e^ *μ*_*m*_ *, μ*_*l*_—mass (cm^2^/g) and linear (1/cm) attenuation coefficient at 46.54 keV [17]

^f^Self-attenuation correction with respect to the water standard (calibration source) is provided to show the scale of self-attenuation effect and is not discussed in the text ^g^ *μ*_*m*_ for Ca

The basic calculations of the *C*_*s*_ */C*_*s*0_ values were performed with Monte Carlo (MC) method; MCNP4C code was used [10]. The MC method provided values of the detector efficiencies *ε*_*s*0_ and *ε*_*s*_ from which *C*_*s*_ */C*_*s*0_ was calculated using Eq. (2). The simulations were performed for the 46.54 keV photons emitted by ^210^Pb. The spectrometric setup [11] for which the calculations were performed comprised a semiconductor detector HPGe (Canberra GX4020) with the resolution 1.9 keV, energy range above 3 keV and relative efficiency 42 %. The detector has a cylindrical shape with the diameter of 6.1 cm and the height of 6.0 cm; the distance between the detector crystal and the 0.5 mm carbon composite window of detector equals 0.6 cm. The detector is covered during measurements with a hood-shaped 1.4 mm thick Teflon protection cap, placed above the detector window; the distance between the detector crystal and the sample is 0.81 cm. The detector is placed in a shielding made of lead bricks 10 cm thick with a 1 mm cadmium and 1 mm copper inner lining.

The simulations were done for the specific measuring geometry where sample containers covered with a cap were positioned axially, directly on the detector. The polystyrene container walls have the density 1.05 g/cm^3^ and the thickness 1 mm. Sample volume is 84.8 cm^3^ with diameter of 6.0 cm, and height of 3.0 cm which is a typical thickness for environmental samples.

The *C*_*s*_ */C*_*s*0_ as a ratio of efficiencies (cf. Eq. 2) is little sensitive to detector model [12, 13]. Therefore, the calculations were performed for nominal detector dimensions provided by the manufacturer. The time of calculations was chosen in order to keep the type A uncertainty [14] of the calculation results less than 0.1 %.

The input data for the computations included the geometry of the measurement and the chemical composition and density of a sample. The simulations were done for four sample natural materials (matrices) with different densities and chemical compositions: peat, water, ash and soil (cf. footnotes to Table 1).

An exemplary calculation procedure is described below for peat (chemical composition: 60 % C, 18.5 % O, 11.5 % Si, 5 % Fe, 3 % Al and 2 % Ca) and element Fe.

**Fig. 1:**
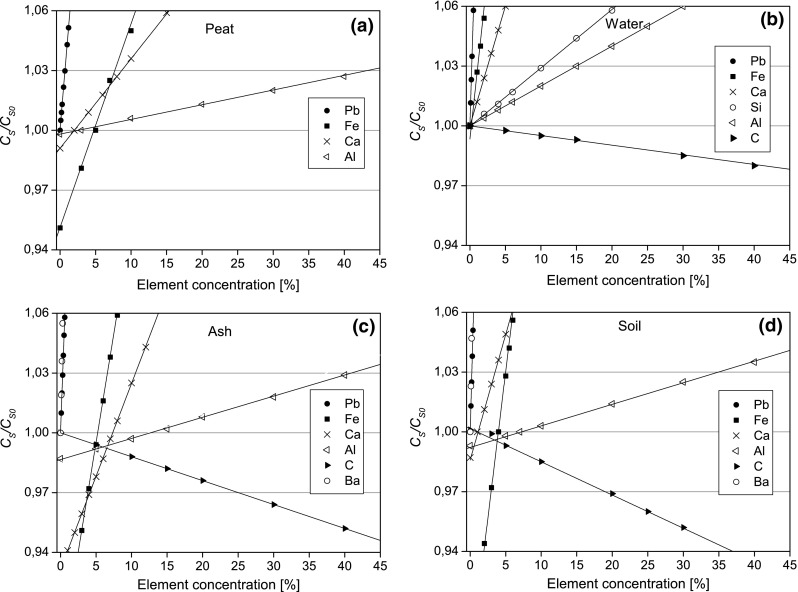
*C*_*s*_ */C*_*s*0_ vs. element concentration for peat (**a**), water (**b**), ash (**c**) and soil (**d**). The threshold concentration is defined by the *C*_*s*_ */C*_*s*0_ ratios equal to 1.03 or 0.97. *Fitting lines* are plotted for clarity

1. *C*_*s*0_ calculations for the above-mentioned baseline peat composition,
2. *C*_*s*_ calculations for peats with Fe concentration changing from 0 to 10 %. In order to compensate for the change in Fe concentration concentrations of the major peat components (C, O, Si) had to be modified proportionally to their original concentrations so that their sum remained equal to 100 %. Concentrations of minor components were not changed. For example, the resulting peat composition for Fe concentration 6 % was: 59.3 % C, 18.3 % O, 11.4 % Si, 6 % Fe, 3 % Al and 2 % Ca.

3. Determination of the relationship between Fe concentration in peat and *C*_*s*_ */C*_*s*0_ value (cf. Fig. 1).

The above calculations were performed for the reference spectrometric setup (detector efficiency 42 %, sample diameter *d * 6.0 cm, sample height *h* 3.0 cm, sample density *ρ* 1.0 g/ccm; coded as GX40_d60h30_ρ1.0). In order to evaluate the applicability range of the obtained results, in particular of the threshold values for element concentrations, additional calculations were performed for ash measured in other spectrometric setups (cf. Table 2):

**Table 2:** Threshold element concentration for ash and for different spectrometric setups

	GX40_d60h30_ρ1.0	GX40_d20h30_ρ1.0	GX40_d100h30_ρ1.0	GX40_d60h10_ρ1.0	GX40_d60h40_ρ1.0	GX20_d60h30_ρ1.0	GX80_d60h30_ρ1.0	GX40_d60h30_ρ0.5	GX40_d60h30_ρ1.5	GX40_d60h40_ρ2.0
C	24 % (0 %)	33 %	21 %	50 %	22 %	25 %	24 %	43 %	20 %	17 %
O	MajEl (48 %)	MajEl	MajEl	MajEl	MajEl	MajEl	MajEl	MajEl	MajEl	MajEl
Al	41 % (13 %)	50 %	38 %	67 %	39 %	42 %	41 %	60 %	35 %	32 %
Si	MajEl (21 %)	MajEl	MajEl	MajEl	MajEl	MajEl	MajEl	MajEl	MajEl	MajEl
Ca, K	*10.6* *%* (7 %)	*11.7* *%*	*10.3* *%*	*13.5* *%*	*10.3* *%*	*10.8* *%*	*10.6* *%*	*13.0* *%*	*9.9* *%*	*9.6* *%*
Fe	*6.7* *%* (5 %)	*7.1* *%*	*6.5* *%*	*8.0* *%*	*6.6* *%*	*6.8* *%*	*6.6* *%*	*7.6* *%*	*6.4* *%*	*6.2* *%*
Pb	0.31 % (0 %)	0.42 %	0.27 %	0.60 %	0.29 %	0.34 %	0.31 %	0.53 %	0.24 %	0.22 %

Explanation of symbols and abbreviations cf. Table 1

^a^GX20, GX40, GX80—detector with the relative efficiency 20, 42, 80 %, respectively

- detectors with the crystal diameters 4.8, 6.1 and 7.6 cm (relative efficiency about 20, 42 and 80 %, respectively—cf. formula in [15], the end-cap diameters 7.6, 7.6 and 9.5, respectively); MCNP models of these detectors are based on MCNP model of GX4020 detector,
- sample diameters *d* 2.0 and 10.0 cm,
- sample heights *h* 1.0 and 4.0 cm,
- sample densities ρ 0.50, 1.5 and 2.0 g/ccm.

## Results and discussion

The relationships between element concentrations and *C*_*s*_ */C*_*s*0_ for the peat, water, ash and soil are presented in Fig. 1a–d. Slopes of the regression lines are the measure of sensitivity of self-attenuation correction *C*_*s*_ to changing concentrations of different elements.

Three groups of elements were identified:

- major elements, determination of their concentrations in the analysed material is indispensable (e.g. C, O and Si for peat),
- elements whose determination in the analysed material is necessary when the expected element concentration in a sample exceeds threshold value; in some cases (see Fe below) also a lower threshold can be defined,
- elements whose determination in the analysed material is not necessary because the threshold concentration is significantly different from the expected concentration of an element in the studied material.

We understand the threshold concentration as the concentration of an element in a sample for which the error of *C*_*s*_ resulting from assuming average concentration of this element in the analysed material, exceeds the maximum acceptable uncertainty. For the maximum acceptable uncertainty of 3 % assumed in this work the threshold values are defined by *C*_*s*_ */C*_*s*0_ = 1.03 or *C*_*s*_ */C*_*s*0_ = 0.97 (cf. Fig. 1).

Table 1 shows classification of the elements and their respective threshold concentrations for the considered materials. E.g. for soil samples:

- Si and O are major elements,
- threshold concentration of Ca, K, Fe equals 4, 4 and 5 %, respectively, whereas concentration of these elements in soil equals 1, 1 and 4 %, respectively. Thus, it is necessary do determine Ca, K and Fe concentrations. Iron is a specific case as two threshold concentrations can be defined, for *C*_*s*_ */C*_*s*0_ = 0.97 and *C*_*s*_ */C*_*s*0_ = 1.03,
- threshold concentration of Al and Pb equal 35 and 0.25 %, respectively, whereas concentration of these elements in soil is significantly lower and equals 7 and 0 %, respectively. Thus, it is not required to determine Al and Pb concentrations,
- threshold concentration of C equals 20 %, whereas concentration of this element in considered soil equals 1 %. Thus, it is required to determine C concentration only for organic soils.

One has to note that for materials of industrial origin also other elements with concentrations exceeding the environmental levels have to be considered. For example, Ba concentrations in drilling wastes reach up to several percent while the threshold concentration estimated for a typical drilling waste is at a fraction of a percent (cf. Fig. 1c–d).

**Fig. 2:**
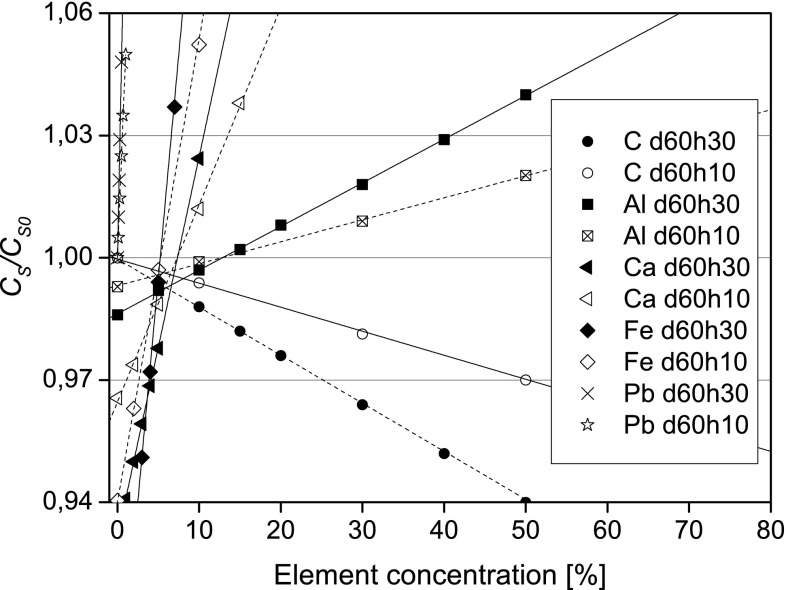
*C*_*s*_ */C*_*s*0_ vs. element concentration for ash and two different spectrometric setup: GX40_d60h30_ρ1.0 and GX40_d60h10_ρ1.0

Results of calculations aiming at determination of applicability range for the obtained threshold values are presented in Fig. 2 and Table 2. Figure 2 shows *C*_*s*_ */C*_*s*0_ values for the reference setup (GX40_d60h30_ρ1.0) and for the setup GX40_d60h10_ρ1.0, that revealed the largest discrepancies from the reference setup. As can be seen, the *C*_*s*_ */C*_*s0*_ for these geometries (and for other analysed setups too) differ by less than 1.5 % for the *C*_*s*_ */C*_*s*0_ values for reference setup from the range 0.97–1.03 and reach up to 3 % for the *C*_*s*_ */C*_*s*0_ values from the ranges 0.94–0.97 and 1.03–1.06. The relative differences of the threshold concentrations between most spectrometric setups do not exceed 20 % (cf. Table 2). Only for the setups GX40_d60h10_ρ1.0 and GX40_d60h30_ρ0.5, for which absorption is significantly smaller than for the reference setup, the *C*_*s*_ */C*_*s*0_ values are overestimated by a few tens of percent.

A crucial, for the applicability of the basic calculations, factor is whether classification of a particular element to one of the three groups (cf. “Results and discussion” section) is the same for different setups. For example, as can be seen from Table 2 classification for ash is the same for all setups, i.e. Ca, K and Fe have to be determined while there is no need to determine Al and Pb.

## Conclusions

Sensitivity of self-attenuation correction *C*_*s*_ to the accuracy of chemical composition analysis was evaluated. The analyses were done by means of Monte Carlo simulation method for a cylindrical samples with 3 cm in height, 6 cm in diameter and for four types of environmental materials: peat, water, ash and soil. For each of these materials the major elements were selected whose determination in the analysed material is necessary. For the remaining elements that often occur in environmental samples, threshold levels of their concentration were determined—if the expected element concentration in a sample exceeds this value, its determination is indispensable, assuming the accuracy of *C*_*s*_ determination at 3 %. The applicability range of the obtained results, in particular of the threshold values for element concentrations, was evaluated.

The results presented in the paper help to limit the scope of chemical analyses in measurements of ^210^Pb activity by gamma-spectrometry to those elements that are essential for correct determination of *C*_*s*_ correction. A proper selection of the elements leads to the reduction of time and resources necessary for a reliable determination of ^210^Pb in environmental samples.
